# A lateral flow immunoassay-based survey reveals a low-frequency truncated Solenopsis invicta venom 2-like protein and unique Solenopsis invicta venom 2 protein genotypes in *Solenopsis invicta*

**DOI:** 10.3389/finsc.2025.1527130

**Published:** 2025-04-07

**Authors:** Steven M. Valles, Robert K. Vander Meer, Alden S. Estep

**Affiliations:** Center for Medical, Agricultural and Veterinary Entomology, United States Department of Agriculture, Agricultural Research Service (USDA-ARS), Gainesville, FL, United States

**Keywords:** Solenopsis invicta, Solenopsis richteri, venom, alkaloids, Formicidae

## Abstract

The purpose of this research was to examine the Solenopsis invicta venom 2 protein and transcript among *Solenopsis invicta* fire ants exhibiting an unusual response to antibody interrogation of this protein. Sequence and phylogenetic analyses combined with Western blotting and lateral flow immunoassay were employed to examine the venom proteins from these fire ants. Genotypic variation was discovered in the Solenopsis invicta venom 2 gene. Many of these unique genotypes exhibited strong identity to the Solenopsis richteri venom 2 ortholog from the congener, *Solenopsis richteri*. Phylogenetic analysis of these sequences revealed a significant evolutionary relationship with *Solenopsis richteri* despite being obtained from *Solenopsis invicta*. A unique, truncated, Solenopsis invicta venom 2-like protein was also discovered in these colonies originating from a unique locus on chromosome 10 where multiple duplication events have apparently copied this gene. These results suggest the possible presence of a cryptic species.

## 1 Introduction

*Solenopsis richteri* Forel and *Solenopsis invicta* Buren were introduced accidentally from South America into the United States at the Mobile, Alabama, port area around 1918 and 1933, respectively (1). *S. richteri* is a more temperate species from southern Argentina and *S. invicta* has subtropical origins in Brazil and northern Argentina. Interestingly, the two species intermate and produce a reproductively viable hybrid (2, 3). Since their introductions, *S. invicta* has greatly expanded its range to include areas from Florida to Virginia and west into California (4), and the species have segregated roughly based on their cold tolerance (*S. richteri* > hybrid > *S. invicta*). Currently, *S. richteri* is largely confined to a contiguous region in northern Mississippi (5), Alabama (6), and Tennessee (7). The hybrid occupies a large band between *S. richteri* to the north and *S. invicta* to the south and is currently the dominant form in Tennessee (7, 8; Oliver et al., In Press)^1^.

Both species and the hybrid possess a potent venom that poses a significant human health risk (9, 10). Among those stung, 5% require medical attention and 2% exhibit serious allergic responses, including anaphylaxis (9, 11, 12). The venom is comprised of a mix of alkaloids (>95%) and more than 46 proteins (0.01%) (13, 14). Four of these proteins have been studied extensively and are well characterized, namely Solenopsis (invicta or richteri depending on the species) venom 1 protein, venom 2 protein, venom 3 protein, and venom 4 protein (3, 15) with the remaining proteins playing undefined roles (13). Solenopsis venom protein 2 comprises the largest proportion (~67%) of these four proteins (10) and each of the parent invasive fire ant species, *S. invicta* and *S. richteri*, expresses an ortholog with 80% identity between the protein sequences (15, 16).

Lateral flow immunoassay tests (InvictDetect™ and InvictDetect Plus™, Agdia, Inc., Elkhart, IN) were developed and commercialized for rapid field identification of *S. invicta*, *S. richteri*, and their hybrid, targeting the Solenopsis venom 2 protein (16, 17). Field evaluations with InvictDetect™ and InvictDetect Plus™ revealed false negative responses among a small number of *S. invicta* colony samples. The objective was to examine the Solenopsis invicta venom 2 protein (henceforth Soli2) in these false negative-yielding *S. invicta* colonies to understand the reason for the failed responses.

## 2 Materials and methods

### 2.1 Lateral flow immunoassay and venom purification

Field evaluations of *S. invicta* fire ants with InvictDetect™ or InvictDetect Plus™ immunostrips were conducted in Gainesville, Florida, and Atlanta, Georgia, from 2015 through to 2020. Colonies yielding three consecutive false negative InvictDetect™ or InvictDetect Plus™ immunostrip responses were taken to the laboratory for additional analysis, including venom purification and Soli2 transcript sequencing (**Table 1**). Two normally responding (to InvictDetect™ or InvictDetect Plus™) ant colonies were included and served as controls, Soli_WtA and Soli_WtB (**Table 1**). These are referred to as “wildtype” *S. invicta*. Venom was partially purified using a modified method of Paterson Fox et al. (18). Briefly, 5 g of worker ants were placed into a 50 ml conical tube containing 5 µl of 12.1 M HCl on the inside cap of the tube for 15 minutes. The acid vapors irritated the ants, inducing the release of their venom. Deionized water (5 ml) and hexane (5 ml) were added to the tube to extract and separate the venom protein components from the alkaloids. The tube was rocked for 5 minutes, centrifuged at 3,000 RPM, and the hexane phase was removed and discarded. Fresh hexane (5 ml) was added to the tube, and the process was repeated two additional times. The aqueous phase was frozen and lyophilized overnight to dry.

**Table 1:** Summary of metadata for fire ant colonies collected and analyzed for this study.

Colony	Species	Date collected	County	State	Latitude	Longitude	Social form	LFIA result
Soli_2	*S. invicta*	10-Nov-15	Alachua	Florida	29.636945	-82.385373	Polygyne	Negative
Soli_3	*S. invicta*	19-Nov-15	Alachua	Florida	29.584938	-82.338512	Monogyne	Negative
Soli_4	*S. invicta*	19-Nov-15	Alachua	Florida	29.584938	-82.338512	Monogyne	Negative
Soli_5	*S. invicta*	2-Dec-15	Alachua	Florida	29.70347	-82.386453	Monogyne	Negative
Soli_7	*S. invicta*	15-Mar-16	Alachua	Florida	29.638376	-82.376375	Monogyne	Negative
Soli_8	*S. invicta*	20-Mar-16	Alachua	Florida	29.644494	-82.331456	Monogyne	Negative
Soli_9	*S. invicta*	20-Mar-16	Alachua	Florida	29.644494	-82.331456	Monogyne	Negative
Soli_10	*S. invicta*	23-Mar-16	Alachua	Florida	29.613575	-82.376574	Monogyne	Negative
Soli_11	*S. invicta*	4-Apr-16	Fulton	Georgia	34.003045	-84.286832	Monogyne	Negative
Soli_12	*S. invicta*	26-Sep-17	Pima	Arizona	32.892	-109.838	Monogyne	Negative
Soli_13	*S. invicta*	4-May-16	Alachua	Florida	29.638357	-82.375694	Monogyne	Negative
Soli_WtA	*S. invicta*	24-Aug-15	Alachua	Florida	29.634393	-82.359095	Monogyne	Positive
Soli_WtB	*S. invicta*	24-Aug-15	Alachua	Florida	29.634393	-82.359095	Monogyne	Positive
Solr_WtA	*S. richteri*	8-Nov-15	Shelby	Tennessee	35.215955	-89.53033	Monogyne	Positive
Solr_WtB	*S. richteri*	8-Nov-15	Shelby	Tennessee	35.215955	-89.53033	Monogyne	Positive
Solg_Wt	*S. geminata*	14-Apr-16	Alachua	Florida	29.65775	-82.472245	Monogyne	Negative

### 2.2 Venom protein molecular detection

Soli2 transcripts were amplified by reverse transcription polymerase chain reaction (RT-PCR) with oligonucleotide primers specific to the conserved 5’ and 3’ termini of the mature Soli2 transcripts (19). Total RNA was extracted from a pooled group (*n* = 10) of worker ants using the TRIzol method (Thermo Fisher Scientific) according to the manufacturer’s directions. RNA was treated with DNAse and used as a template for two-step RT-PCR.

Briefly, 0.5 µl (10-50 ng) of the DNAse-treated total RNA was mixed with 10 mM dNTPs and 1 µM reverse oligonucleotide primer CS5 (5’ TTATATGCACAATATTTTATTGTTAACCCAACAC), heated to 65°C for 5 minutes, and then placed on ice for 1 minute. To this mixture, 5x first strand buffer and Superscript Reverse Transcriptase (RT, Life Technologies) were added, and the reaction was incubated at 55°C for 30 minutes. The RT reaction was terminated by incubation at 70°C for 15 minutes. The cDNA was used as a template for PCR with oligonucleotide primers 4CS (5’ GTTAAATGAATAAAGTCACTCATACAACTTCTCTA) and 5CS under the following cycling conditions: 94°C for 2 minutes; 35 cycles of 94°C for 15 seconds and 55°C for 15 seconds; and 68°C for 1.5 minutes and 1 cycle of 68°C for 5 minutes. Amplicons were purified on 0.75% Agarose gels and visualized with SYBR Safe (Life Technologies). Amplicons were ligated into the pCR4-TOPO vector (Life Technologies) and transformed into TOP10 *E. coli* competent cells (Life Technologies). Bacterial colonies were picked and inoculated in Luria broth with ampicillin (75µg/ml), incubated at 37°C while shaking at 225 rpm, and positively determined for the presence of the insert by PCR with the original oligonucleotide primers.

Insert-positive plasmid DNA was purified using the QIAprep Miniprep Spin kit (Qiagen) and clones (a minimum of 5 per colony) were sequenced by the Sanger method. Consensus sequences for each clone were used in subsequent analyses.

5’ Random amplification of cDNA ends (RACE) was conducted on RNA isolated from several colonies expressing the Soli2 truncated venom transcript to confirm the size of the transcript. cDNA was synthesized with a gene-specific oligonucleotide primer (5CS), and the PCR was subsequently completed with a nested gene-specific primer (8CS, 5’ GGCCTTGAGTCTCTCTATCTACACATCTAGAA) and the GeneRacer Abridged Anchor Primer. The anticipated size of the amplicon was compared with one empirically derived.

Colony social form (monogyne and polygyne) was determined by detection of the *Gp-9* alleles as previously described (20).

### 2.3 Cytochrome oxidase and mitochondrial genome sequencing

The mitochondrial cytochrome oxidase I gene from a subset of the *S. invicta* colonies (Soli_3, Soli_4, Soli_7, Soli_10, Soli_11, and Soli_WtA) was amplified, cloned, and sequenced. The PCR was conducted using total DNA extracted from a pooled group of 10 worker ants as a template. The universal oligonucleotide primers LCO1490 (5’ggtcaacaaatcataaagatattgg) and HCO2198 (5’taaacttcagggtgaccaaaaaaatca) reported by Folmer et al. (21) were used. Cloning and Sanger sequencing were completed as described above.

The mitochondrial genomes of colonies Soli_3, Soli_7, Soli_WtA, and Soli_WtB were sequenced using nanopore R10 sequencing chemistry. Total DNA from each colony was end prepped and repaired using NEBNext FFPE Repair Mix and Ultra2 End Prep Mix (New England Biolabs) following the genomic DNA ligation sequencing protocol (SQK-NBD114.96, Protocol: NBE_0171_v114_revO_15Sep2022, Oxford Nanopore Technologies, Oxford, UK). After the initial end prep, subsequent steps were conducted using the amplicon sequencing protocol (SQK-NBD114.96, Protocol: NBA_9170_v144_revM_15Sep2022, Oxford Nanopore Technologies). Barcoded samples were sequenced on a MinION Mk1B with an R10.4.1 flow cell (Oxford Nanopore Technologies) using MinKNOW software. The sequencing was conducted for 72 hr. Reads were binned and basecalled automatically by the MinKNOW software using a minimum quality cutoff of 10. Raw sequencing data for each sample has been deposited in NCBI under BioProject: PRJNA1179381.

Assembly and indexing of each mitochondrial genome were conducted by mapping to the *S. invicta* mitochondrial genome (NCBI Accession: NC014672.2) using Minimap2 (22). The resulting binary assembly files were displayed in IGV to examine the coverage and the consensus option was used to generate a final consensus for each sample (23).

### 2.4 Protein separation and Western blotting

To determine whether the truncated transcript identified by molecular analysis was being translated into protein, Western analysis was conducted against the purified worker ant venom from ant colonies Soli_4, Soli_11, Soli_WtA, and Soli_WtB. Venom proteins were applied (7.5 µg) and separated on a 4%–20% gradient SDS-PAGE gel. Gel-separated proteins were electroblotted onto PVDF membranes and blocked in TBS (tris buffered saline; 20 mM Tris-HCl, 500 mM NaCl, pH 7.5) + 1% BSA (bovine serum albumin) for 2 hours. Blots were probed with monoclonal antibody (mAb) 6A8D7 [prepared to a 15 amino acid sequence (NKELKIIRKDVAECL) corresponding to positions 2–16 of the Soli2 venom protein], mAb 3H6B9 [corresponding to a fourteen amino acid sequence (IEAQRVLRKDIAEC) at positions 2–15 of the Soli2 venom protein] (16), or mAb 7D7F7 [corresponding to a 17 amino acid sequence (NPDPAVIKERSMKMCTK) at positions 48–64 of the Soli2 venom protein]. Monoclonal antibodies were produced by ProMab Biotechnologies Incorporated (Richmond, CA) using the hybridoma method. Monoclonal antibody in concentrations normalized to *S. invicta* (6A8D7 = 1.6 µg/ml; 3H6B9 = 1.8 µg/ml; 7D7F7 = 0.5 µg/ml) was added to the TBS + 1% BSA solution containing the blot for 2 hours at room temperature with shaking (40 rpm). Membranes were rinsed twice with TTBS (TBS + 0.05% Tween 20), probed with secondary antibody (0.1 μg/ml), goat anti-mouse conjugated with alkaline phosphatase (Sigma, St. Louis, MO) for 1 hour, and rinsed twice with TTBS. The membrane was incubated for several minutes with BCIP (5-bromo-4-chloro-3-indolyl-phosphate) and NBT (nitro blue tetrazolium) for the colorimetric detection of alkaline phosphatase activity. Once bands were detected on the blot, the reaction was terminated by rinsing the membrane with deionized water.

### 2.5 Alkaloid analysis

Worker ants from *S. invicta* colonies Soli_9, Soli_11, and Soli_13 were qualitatively analyzed for their venom alkaloids and compared with a standard *S. invicta* colony. Chromatograms were obtained from an Agilent Intuvo 9000 GC system (Santa Clara, CA) equipped with an HP-5 MS ultra-inert nonpolar column, 30 m × 0.25 m i.d. column, coupled to a 5977 B mass spectral detector and a MassHunter Data Acquisition Workstation version 10.0.368 (Santa Clara, CA). The injector temperature was set at 250°C. The oven temperature was programmed at 40°C for 2 min and then to 285°C at 5°C/min, followed by a 10 min hold at 285°C.

The piperidine alkaloids were identified by their base peak fragment at m/z 98. The alkaloid composition of *S*. *invicta* has been known for decades due to its importance as an invasive species throughout the southeastern U.S.A. The four major *S. invicta* worker alkaloids are *trans*-piperidines: 2-methyl-6-tridecyl-piperidine (compound 2), 2-methyl-6-tridecenyl-piperidine (compound 3), 2-methyl-6-pentadecyl-piperidine (compound 4), and 2-methyl-6-pentadecenyl-piperidine (compound 5). In addition, there are minor amounts of 2-methyl-6-undecyl-piperidine (compound 1) and 2-methyl-6-heptadecenyl-piperidine (compound 6). The component peak heights are proportional to the relative amounts of each component. The variation in peak ratios in the samples was within normal observed variation in *S. invicta* samples.

### 2.6 Phylogenetic analysis

Evolutionary relationships among Soli2 venom protein sequences and *S. invicta* cytochrome oxidase I sequences were inferred using the maximum likelihood method and JTT matrix-based model (24). Phylogenetic trees with the highest log likelihood were used for the analysis. The percentage of trees in which the associated sequences clustered together was calculated with 500 bootstrap iterations. The initial trees for the heuristic search were obtained automatically by applying the Neighbor-Join and BioNJ algorithms to a matrix of pairwise distances estimated using the JTT model, and then selecting the topology with the superior log likelihood value. Trees are drawn to scale, with branch lengths measured in the number of substitutions per site. Fourteen Soli2 sequences with a total of 138 positions comprised the final dataset for the Soli2 venom proteins and eight sequences with a total of 449 positions comprised the final dataset for cytochrome oxidase I. Evolutionary analyses were conducted in MEGA11 (25). A similar evolutionary analysis was conducted on the mitochondrial genome sequences but with the use of the Kimura Two Parameter model on a matrix of 5 sequences by 15,549 positions (26).

### 2.7 Genome/sequence alignments

Soli2 venom protein transcript sequences were aligned against the *Solenopsis invicta* reference genome (UNIL_SINV_3.0, GCA_016802725.1) using the Splign alignment tool (27) to identify the locus, expressed (exons), and intervening sequences (introns). Locus assignment was made based on the highest exon identity observed for a given sequence. Introns were expressed as the number of nucleotides comprising the sequence.

The open reading frames (ORFs) predicted for the Soli2 and Soli2-like translated sequences were aligned using ClustalW in the Vector NTI Advance 11.5 (AlignX) software package.

### 2.8 Data availability

The sequences obtained throughout the study were deposited into GenBank. Accession numbers assigned to Solenopsis venom 2 proteins from *S. invicta, S. richteri*, and *S. geminata* are listed in **Table 2**. Cytochrome oxidase sequence accession numbers include Si_COI_WtA (PQ038572); Si_COI_3 (PQ038575); Si_COI_4 (PQ038574); Si_COI_7 (PQ038573); Si_COI_10 (PQ038577); and Si_COI_11 (PQ038576). Nanopore sequences were deposited into GenBank under Bioproject PRJNA1179381 (Soli_WtA: SAMN44490061; Soli_WtB: SAMN44490062; Soli_3: SAMN44490063; Soli_7: SAMN44490064).

**Table 2:** Solenopsis invicta venom 2 protein loci identified by alignment of the transcript sequence against the *Solenopsis invicta* reference genome (UNIL_SINV_3.0, GCA_016802725.1) using the Splign alignment tool (27).

Designation	GenBank Accession	Locus*	Sub-Locus	Exon (% identity)					Intron (nts)			
				E1	E2	E3	E4	E5	I1	I2	I3	I4
Soli2_10	PQ040596	105205300	A	98	98	100	99	92	12977	110	452	115
Soli2_4	PQ040601	105205300	A	95	100	100	99	92	12977	110	452	115
Soli2_7	PQ040599	105205300	A	98	98	100	99	92	12977	110	452	115
Soli2_8	PQ040598	105205300	A	97	98	100	99	92	12977	110	452	115
Soli2_9	PQ040597	105205300	A	97	98	100	99	92	12977	110	452	115
Soli2_3	PQ040600	105205300	B	95	98	100	99	92	7128	110	452	115
Soli2_11	PQ040595	105205300	C	98	94	94	91	96	2163	110	453	115
Soli2_13	PQ040591	105205300	C	98	100	100	99	97	2163	110	453	115
Soli2_WtA	PQ040602	105205300	C	98	100	100	99	100	2,404	110	466	115
Soli2Tr_10	PQ041972	105193315		97	98	99	100		2383	450	115	
Soli2Tr_11	PQ041971	105193315		98	98	99	99		2383	450	115	
Soli2Tr_12	PQ041970	105193315		98	98	99	100		2383	450	115	
Soli2Tr_13	PQ041968	105193315		98	100	99	99		2383	450	115	
Soli2Tr_2	PQ041969	105193315		98	98	99	100		2383	450	115	
Soli2Tr_3	PQ041977	105193315		98	98	99	100		2383	450	115	
Soli2Tr_4	PQ041978	105193315		98	98	99	100		2383	450	115	
Soli2Tr_5	PQ041976	105193315		98	98	99	100		2383	450	115	
Soli2Tr_7	PQ041975	105193315		98	100	99	100		2383	450	115	
Soli2Tr_8	PQ041974	105193315		98	100	99	100		2383	450	115	
Soli2Tr_9	PQ041973	105193315		98	100	99	99		2383	450	115	
Solr2_WtA	PQ040594	NA^§^										
Solr2_WtB	PQ040593	NA^§^										
Solg2_Wt	PQ040592	NA^§^										

**Figure 1:**
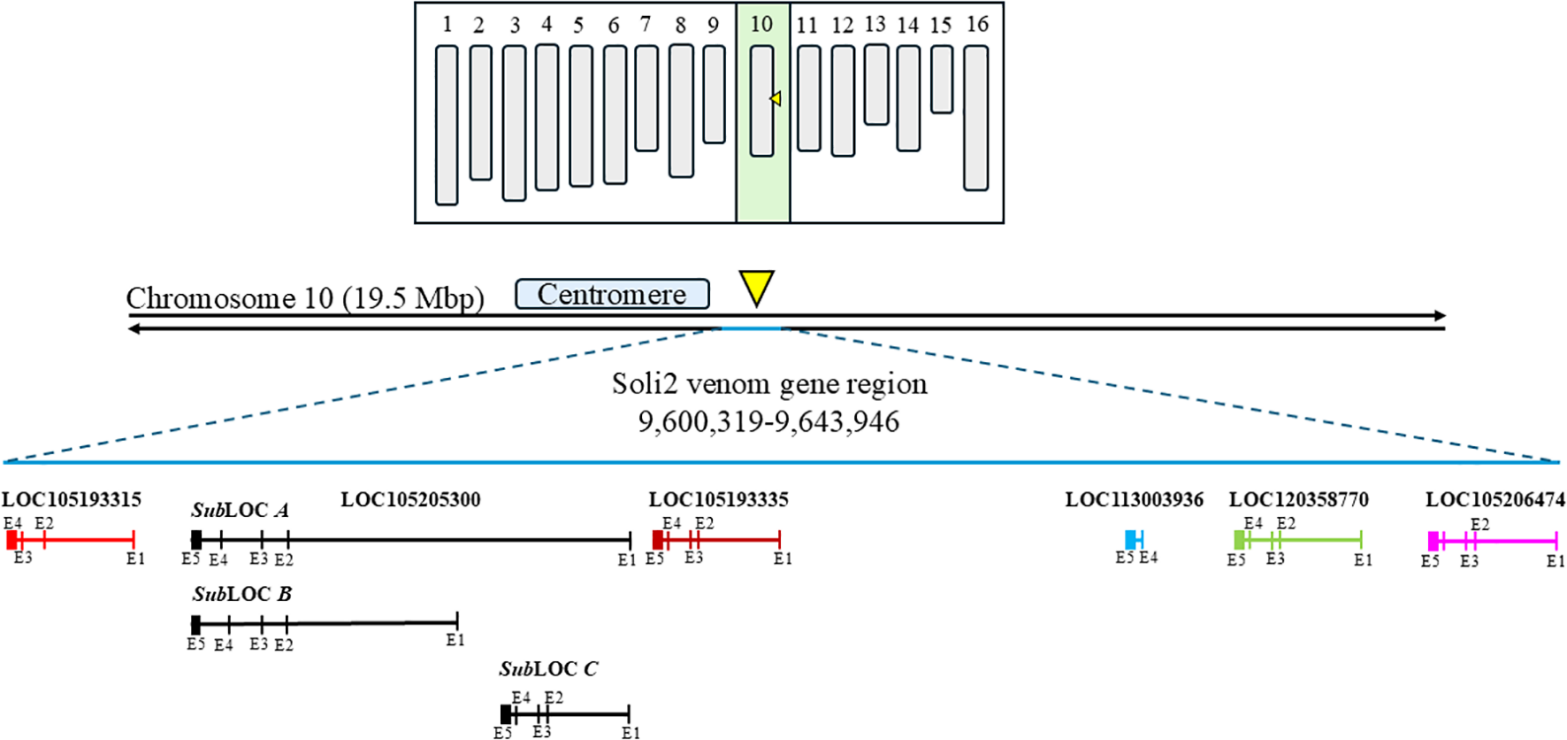
Diagrammatic karyotype of *Solenopsis invicta* identifying the contiguous region on chromosome 10 where Solenopsis invicta venom 2 protein genes reside (yellow arrowhead). The two black horizontal lines represent chromosome 10. The approximate location of the centromere is shown and the Solenopsis invicta venom 2 protein gene region and loci are identified in bold font. Solenopsis invicta venom 2 protein genes are represented by various colored horizontal lines. Exons are approximated and illustrated by vertical lines through the identified locus.

Identity for expressed sequences (exons) is presented as a percentage of identical nucleotide sequences. Intervening sequences are expressed as the number of nucleotides comprising the intron. Graphical representations of each locus and its relative position on chromosome 10 are presented in **Figure 1**.

*Locus as identified on the *Solenopsis invicta* reference genome in GenBank (accession: UNIL_SINV_3.0 GCA_016802725.1).

§ There is no reference genome sequence for either *S. richteri* or *S. geminata*.

## 3 Results

Among the 861 evaluations of verified *Solenopsis invicta* colonies with the InvictDetect™ and/or InvictDetect Plus™ lateral flow immunoassay kits, 12 false negatives (1.39%) for *S. invicta* occurred that did not resolve despite repeated evaluations and increasing the number of pooled worker ants used in the assays. Worker ants from 11 of these colonies were collected and evaluated by RT-PCR for the presence of the Soli2 venom protein transcript. Eight colonies (i.e., Soli_3, _4, _7, _8, _9, _10, _11, _and 13) produced two amplicons [one of 496 nts (short transcript) and one of 596 nts (long transcript)]. Three colonies (Soli_2, _5, and _12) produced a single 496 nt amplicon (short transcript). BLAST analysis (28) of the short and long amplicons revealed similarity to the *Soli2* gene (Genbank accession L09560.1). When the nucleotide sequences were aligned to the *Solenopsis invicta* reference genome, they mapped to a 43,627-nucleotide region of the minus strand of chromosome 10 where *Soli2* resides, specifically the region encompassed by nucleotides 9,600,319 to 9,643,946 (**Figure 1**). Within this region, multiple copies and variations of *Soli2* are present; six *Soli2* loci occupy this region of chromosome 10 (**Figure 1**), including loci 105206474, 120358770, 113003936, 105193335, 10520533, and 105193315. The long transcripts (596 nt) all mapped to locus 105205300 (**Table 2**), but the exons are spread across this locus in multiple copies and locations. Thus, this locus can be thought of as being comprised of three “sub” loci, which we refer to as A, B, and C (**Figure 1**). Five exons comprise the open reading frame (ORF) of the long transcript. Sub-loci A and B share positions for exons 2, 3, 4, and 5, but multiple copies of exon 1 are found upstream. Sub-locus C is found near the 5′ end of the locus, and exons 2, 3, 4, and 5 have different positions and sequences from sub-loci A and B. Another difference between the transcripts generated at each sub-locus was the size of intron 1, which ranged from 2,163 to 12,977 nts (**Table 2**). The remaining introns (2, 3, and 4) were consistent in size (**Table 1**). The original published nucleotide sequence for *Soli2* (Genbank accession L09560.1) was identical to the transcript from colony Soli_WtA (Genbank accession PQ040591), which was collected in 2015 (**Table 1**) and produced a positive response with InvictDetect™.

The short transcripts (496 nts) all mapped to locus 105193315 and were comprised of only four exons. The sequences of exons 1, 2, 3, and 4 of locus 105193315 exhibited sequence similarity with exons 1, 3, 4, and 5 of locus 105205300, respectively. The sequence of exon 2 of locus 105205300 is absent in locus 105193315. Loss of exon 2 (100 nts) in the duplicated locus 105193315 results in a shift of the start codon of the nascent transcript it generates, yielding a 5′-proximally truncated ORF [as compared with the ORF generated from the other (5-exon-containing) *Soli2* paralogs].

**Figure 2:**
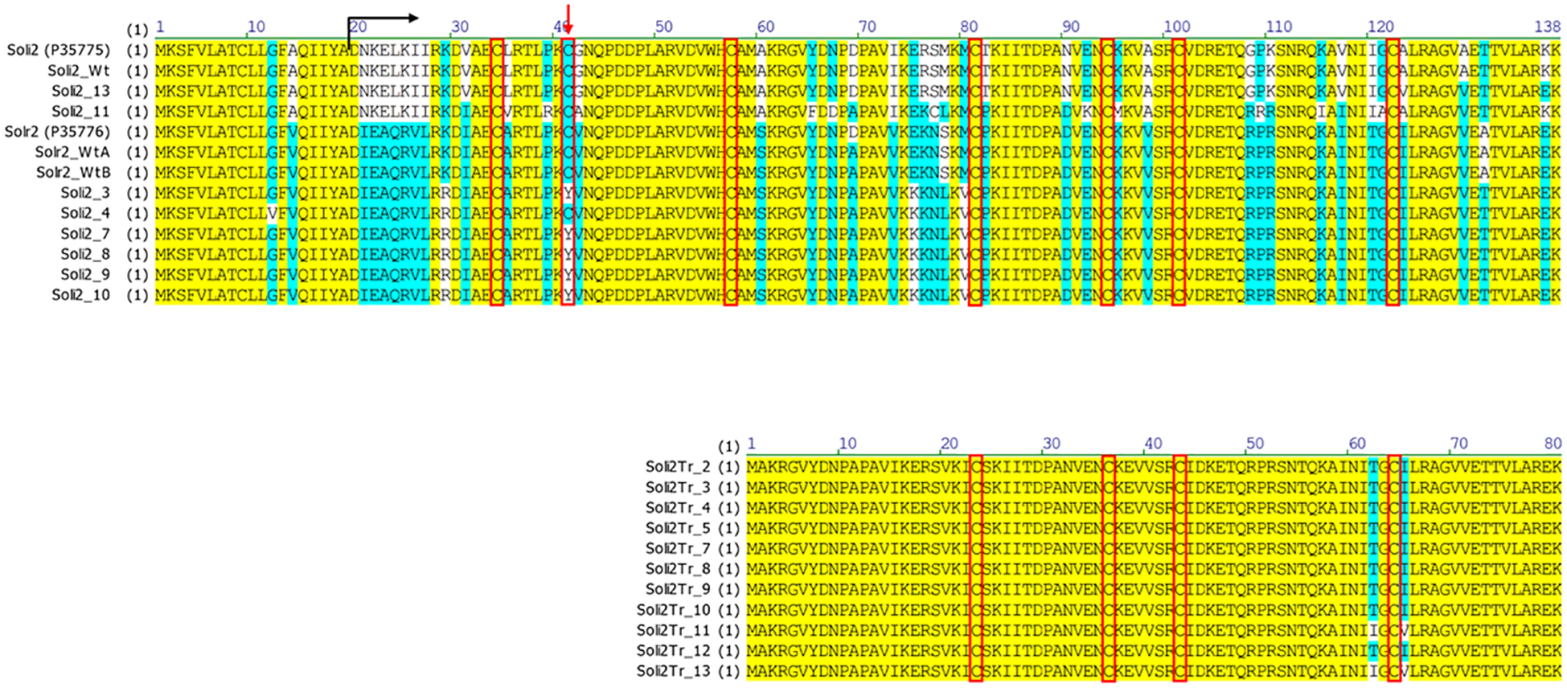
Predicted protein sequences of long (upper) and short (lower) nascent transcripts from *S. invicta* colonies failing an InvictDetect™ assay {compared with normal, or wildtype, Solenopsis invicta venom 2 protein [Soli2 (P35775) and Soli2_Wt] and Solenopsis richteri venom 2 protein [Solr2 (P35776), Solr2_WtA, and Solr2_WtB]}. The black arrow indicates the position of the mature protein and the red arrow indicates the position of the cysteine residue involved in the dimerization of the monomers. Red boxes call out the cysteine positions.

**Figure 3:**
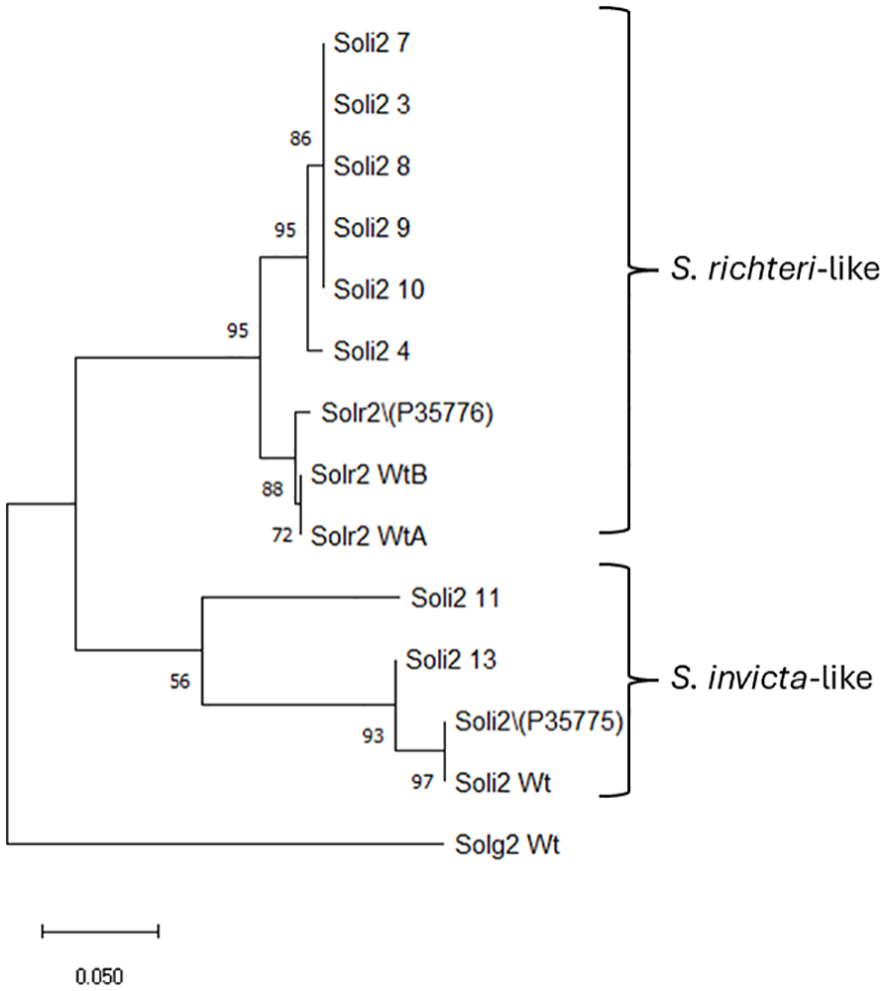
Phylogenetic analysis of Solenopsis venom 2 protein sequences (long sequence) by the maximum likelihood method. Trees are drawn to scale, with branch lengths measured in the number of substitutions per site, 500 bootstrap iterations, and branch support provided as a percentage. Fourteen Soli2 sequences with a total of 138 positions comprised the final dataset for the Soli2 venom proteins. Sample names are provided in **Table 2**.

**Figure 4:**
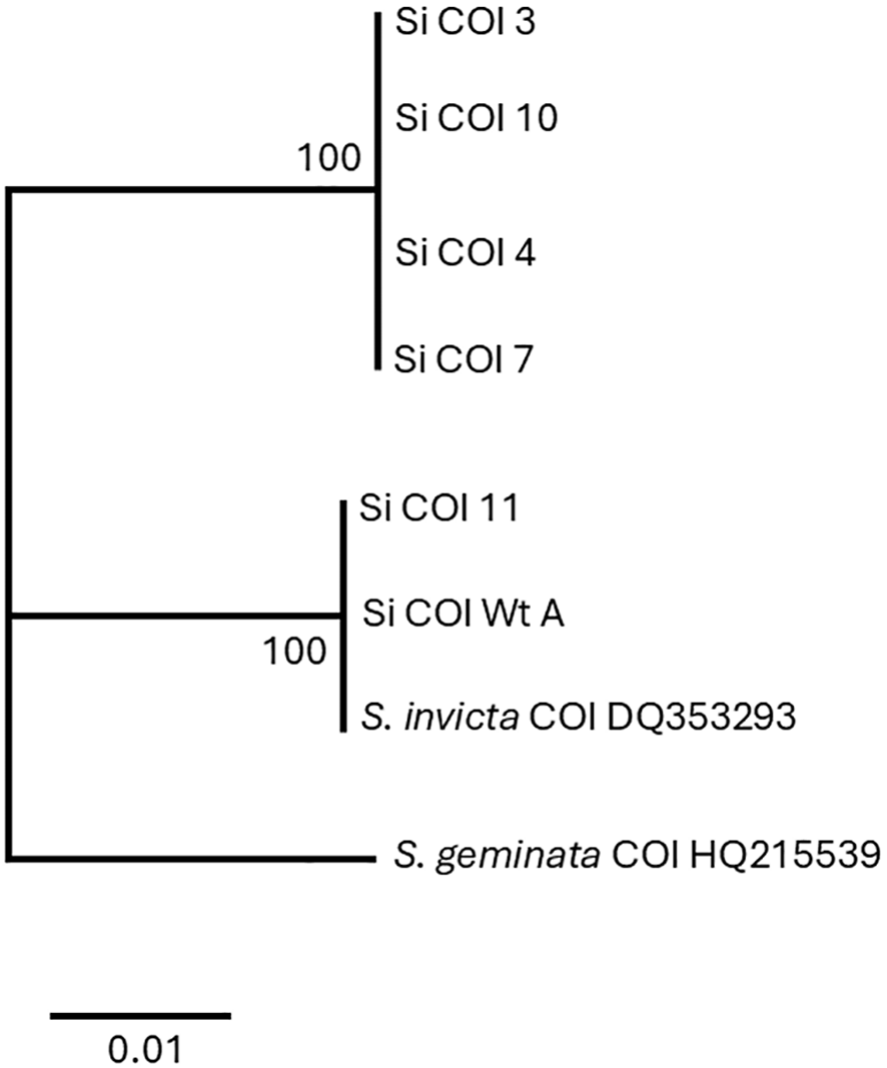
Phylogenetic analysis of cytochrome oxidase I nucleotide sequences by the maximum likelihood method. Trees are drawn to scale, with branch lengths measured in the number of substitutions per site, 500 bootstrap iterations, and branch support provided as a percentage. Eight sequences with a total of 449 positions comprised the final dataset.

Amino acid sequences from the long transcript group of ant colonies that failed to be detected with the Invictdetect™ kit [596 nts, 138 amino acids (aa)] were unique and distinct from the Soli2 venom protein published previously (Genbank accession L09560.1 and P35775) and from our *S. invicta* control colonies (Soli_WtA and Soli_WtB) (29). Alignments of the translated ORF from the long transcript show significant divergence from Soli2 (P35775) in sequence identity (**Figure 2**), which is illustrated in the phylogenetic analysis (**Figure 3**). Two sequences, Soli2_11 and Soli2_13, from ant colonies Soli_11 and Soli_13, respectively, associated most closely with the original Soli2 sequence (P35775). Conversely, sequences Soli2_3, _4, _7, _8, _9, and _10 formed a unique clade most closely aligned with Solenopsis richteri venom 2 protein (Solr2; GenBank accession P35776) from *S. richteri* (**Figure 3**). Notice that the region near the N-terminus of the venom 2 protein sequences is identical to Solr2 (i.e., IEAQRVL) and distinct from Soli2 (NKELKII). Sequences from *S. invicta* colonies Soli2_3, _4, _7, _8, _9, and _10 mapped to sub-locus A and B (LOC105205300), while those with the *invicta*-like NKELKII N-proximal sequence mapped to sub-locus C (**Figure 2**). This divergence was mirrored in a phylogenetic analysis of the cytochrome oxidase I sequences from a limited number of these colonies (**Figure 4**). Notice that cytochrome oxidase I sequences from colonies Soli_3, _4, _7, and _10 (*richteri*-like sequence IEAQRVL) similarly formed a distinct grouping separate from the *S. invicta* (DQ353293) cytochrome oxidase I sequence.

**Figure 5:**
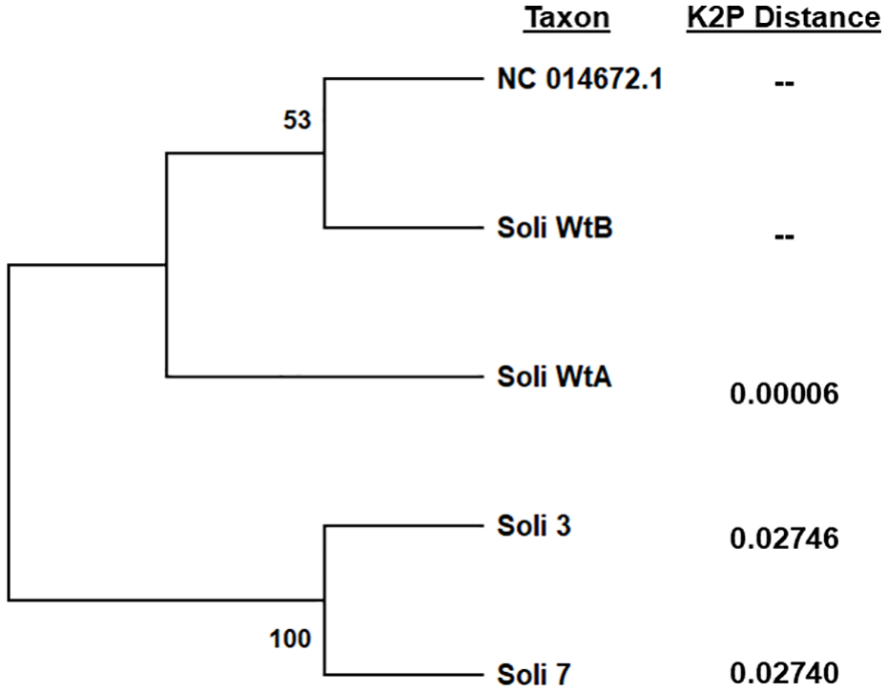
Phylogenetic analysis of complete mitochondrial genome sequences by the maximum likelihood method. Trees are drawn to scale, with branch lengths measured in the number of substitutions per site, 500 bootstrap iterations, and branch support provided as a percentage. Kimura 2-parameter distances were calculated relative to *S. invicta* mitochondrial genome accession NC014672.2. Five sequences with a total of 15,549 positions comprised the final dataset.

Full mitochondrial genomes were assembled after nanopore sequencing of Soli_WtA, Soli_WtB, Soli_3, and Soli_7. Comparison to the *S. invicta* mitochondrial genome (NC014672.2) revealed greater than 99.99% nucleotide identity for Soli_WtA and Soli_WtB (**Figure 5**). In contrast, Soli_3 and Soli_7 had approximately 97.3% nucleotide identity. Phylogenetic reconstruction showed topology similar to that recovered from the COI analysis (**Figures 4**, **5**).

**Figure 6:**
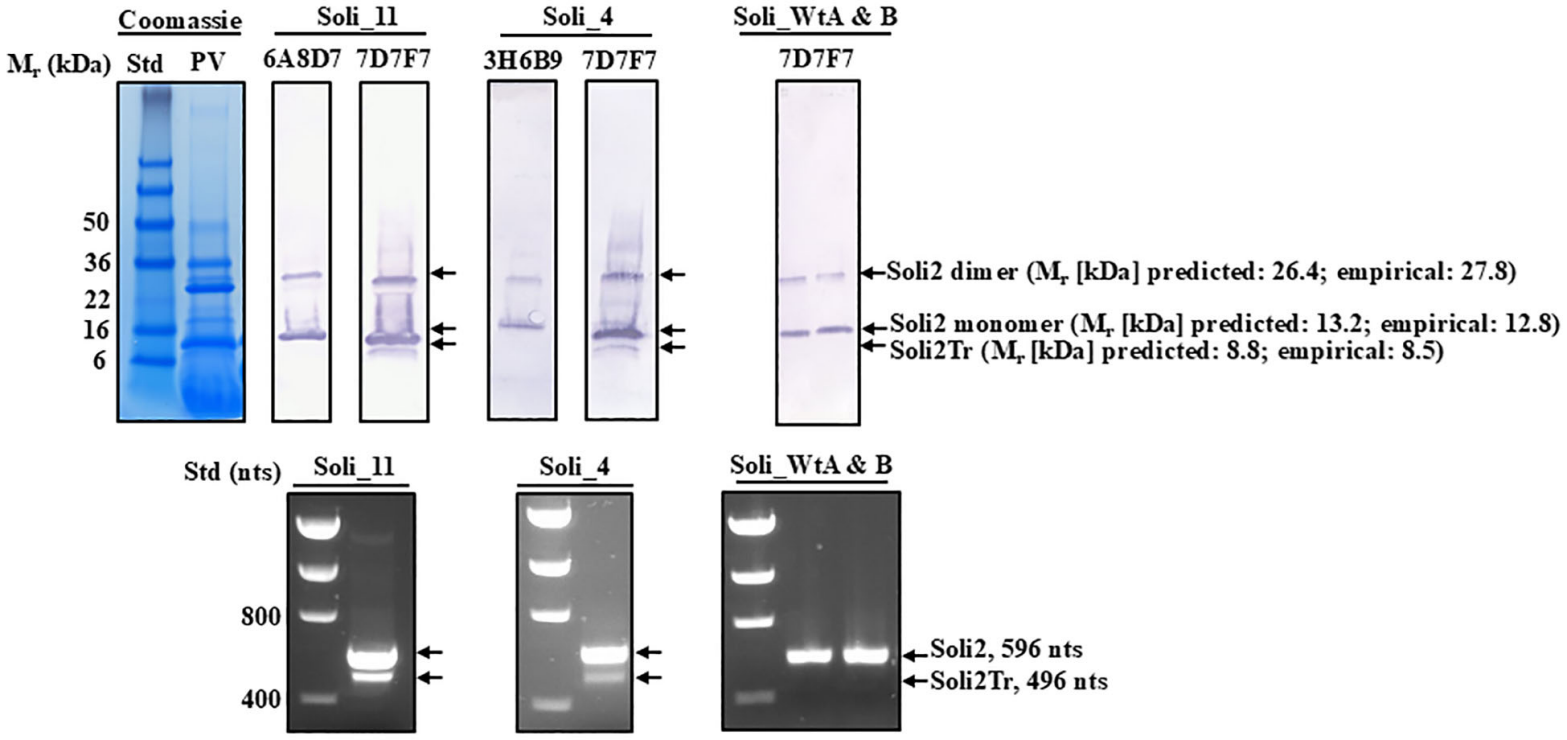
Western blot analysis of purified preparations of venom proteins from worker ants from *S. invicta* colonies Soli_4, Soli_11, Soli_WtA, and Soli_WtB probed with different monoclonal antibody preparations to specific regions of Soli2 and Solr2. Positions and molecular weights of the monomer and dimer of Soli2 and truncated versions of Soli2-like venom proteins are provided on the right. Molecular weight standards (kDa) are shown on the left. The lower panel set shows the amplicons produced for each of the colonies identified in the Western blot directly above. Molecular standards are on the left side and indicate the number of nucleotides.

Amino acid sequence alignments of the short transcript ORFs (**Figure 2**) showed complete conservation among sequences Soli2Tr_2, Soli2Tr_3, Soli2Tr_4, Soli2Tr_5, Soli2Tr_7, Soli2Tr_8, Soli2Tr_9, Soli2Tr_10, and Soli2Tr_12. Two amino acid differences from this group were observed in Soli2Tr_11 and Soli2Tr_13 (**Figure 2**). Translation of these truncated proteins was confirmed by Western blotting of colonies Soli_4 and Soli_11, which was not observed among *S. invicta* ant colonies, yielding a positive result for InvictDetect™ (i.e., Soli_WtA & B; **Figure 6**). Soli2Tr proteins have a predicted molecular weight of 8.8 kDa (**Figure 2**). In purified venom protein preparations from ant colonies Soli_4 and Soli_11 exhibiting the truncated transcript, an 8.5 kDa protein was detected (**Figure 6**).

**Figure 7:**
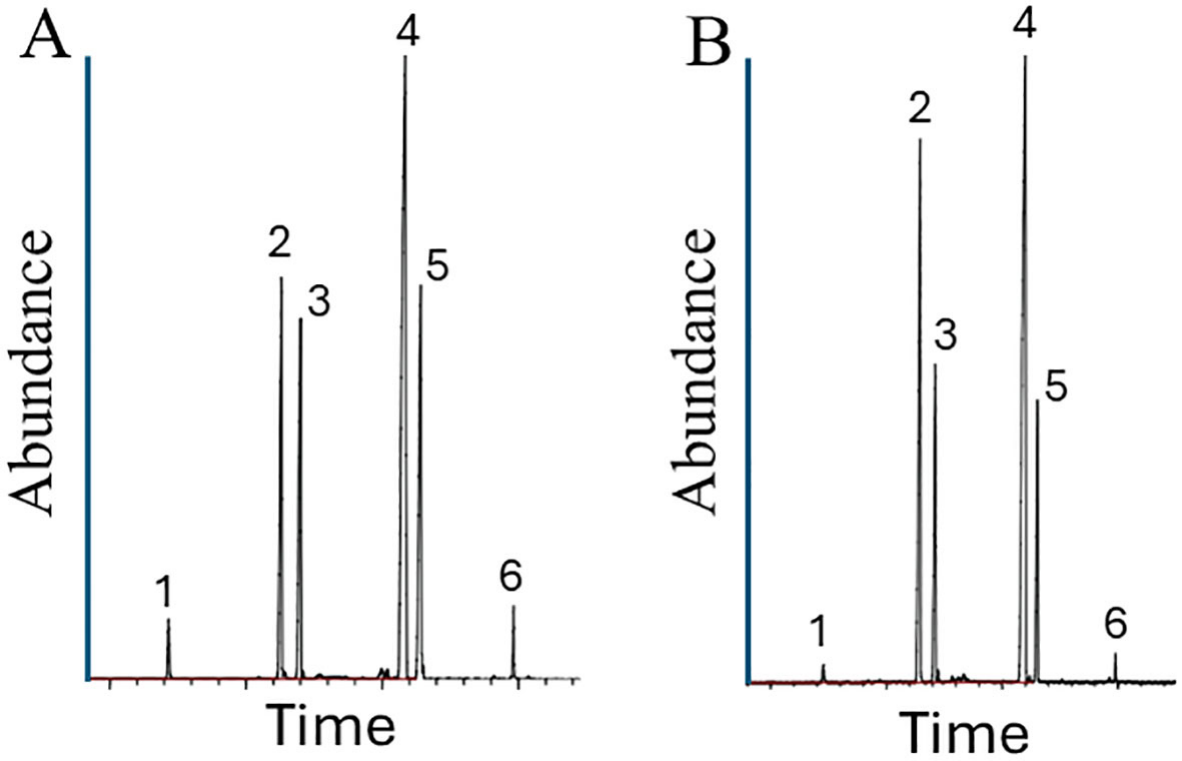
Venom alkaloid analysis of worker ants from *S. invicta* colonies Soli_9 **(A)** and Soli_11 **(B)**. The four major *S. invicta* worker alkaloids are *trans*-piperidines: 2-methyl-6-tridecyl-piperidine (compound 2), 2-methyl-6-tridecenyl-piperidine (compound 3), 2-methyl-6-pentadecyl-piperidine (compound 4), and 2-methyl-6-pentadecenyl-piperidine (compound 5). In addition, there are minor amounts of 2-methyl-6-undecyl-piperidine (compound 1) and 2-methyl-6-heptadecenyl-piperidine (compound 6).

Venom alkaloid chemical analysis of Soli_9, Soli_11, and Soli_13 revealed a variation in peak ratios consistent with *S. invicta* (**Figure 7**).

## 4 Discussion

The lateral flow immunoassays to discriminate and identify the invasive fire ant species, *Solenopsis invicta* and *Solenopsis richteri* (and their hybrids), target the Solenopsis (invicta and richteri) venom 2 protein (16, 17). Monoclonal antibodies are used in tandem to capture and report the presence of these proteins from each ant species. Extensive testing of InvictDetect™ and InvictDetect Plus™ against *S. invicta* nests identified a very small proportion of colonies (1.39%) that yielded unexplained false negative results; the ants were taxonomically identified as *S. invicta* (30) but failed to give a response from the venom-specific antibodies. Nucleotide sequences obtained from Soli2 venom protein transcripts from these ant colonies revealed unique sequences distinct from the sequences used in the development of the immunoassay and originally published decades ago (29, 31, 32). Additionally, a newly discovered truncated Soli2-like protein (Soli2Tr) was identified in these InvictDetect™-non-responding colonies. Initially, we considered the possibility that the truncated Soli2-like transcripts were generated by alternative splicing at locus LOC105205300 on the *S. invicta* reference genome (Genbank accession UNIL_Sinv_3.0). However, this does not appear to be the case. The Soli2 truncated transcripts are not alternatively spliced versions but are transcribed at a unique locus (LOC105193315) upstream of LOC105205300 on chromosome 10, where Soli2 has obviously undergone multiple duplication events. Thus, the InvictDetect™ false negative results may be explained by 1) alteration of the carboxyl-proximal epitope (mAb reporting location) as in colony Sinv_11; 2) alteration of the amino-proximal epitope (mAb capture location) as in colonies Sinv_3, Sinv_4, Sinv_7, Sinv_8, Sinv_9, Sinv_10, and Sinv_11; and 3) complete elimination of the amino-proximal epitope (truncated Soli2-like venom protein) as in colonies Sinv_2, Sinv_5, and Sinv_12. Colony Sinv_13 produced two transcripts, one truncated (Soli2Tr_13) and one identical to the normal, or wildtype, Soli2 (P35775) sequence from LOC105205300. InvictDetect™ failure against this colony may have occurred because of a low expression of Soli2_13 (below InvictDetect™ detection levels) and/or antibody competition with Soli2Tr_13. While the sequence recognized by the reporting antibody in the carboxyl-proximal region on the truncated Soli2 protein is intact, the sequence in the amino-proximal region recognized by the reporting antibody is completely missing, which renders the InvictDetect™ and InvictDetect Plus™ immunoassay ineffective because the missing region prevents the capture of the protein from occurring. Translation of the truncated Soli2 transcript does appear to take place, as a protein of the predicted size was detected by Western analysis in colonies Soli_4 and Soli_11 (**Figure 6**). However, wildtype ant colonies do not appear to express the truncated version from locus 105193315. Thus, multiple variants of Soli2 have been identified, and a new, truncated Soli2-like protein has been discovered. A truncated version of venom 2 protein was also reported from the *S. invicta* x *S. richteri* hybrid ants in Tennessee (19), which was described as Solh2Tr97. In contrast to the *S. invicta* truncated versions of Soli2, Solh2Tr97 is an alternatively spliced version (19).

An interesting, and perplexing, aspect of these sequence variations for the Soli2 long transcript is the evolutionary relationship between the two invasive species of ants, *S. invicta* and *S. richteri*. All of the colonies discovered by false negative immunoassay tests in this study were identified morphologically as *S. invicta* (30), collected from areas known to be populated exclusively by *S. invicta* (1), and exhibited venom alkaloid profiles consistent with *S. invicta* (2, 33). However, phylogenetic analysis of the venom 2 protein sequence predicted from six of these *S. invicta* colonies appeared more closely related to the venom 2 ortholog from *S. richteri* (i.e. Solr2 P35776) than *S. invicta* (Soli2 P35775) (**Figure 3**). Indeed, the specificity of the InvictDetect™/InvictDetect Plus™ to capture monoclonal antibodies for each species resides in the unique amino-proximal sequence of the venom protein of each species and was chosen for the immunoassay because of this significant difference. Note that sequence NKELKII (aa position 21) was originally described from *S. invicta* (P35775) and amino acid sequence IEAQRVI (also at aa position 21) was described from *S. richteri* (P35776) (**Figure 2**). Until the present study, the IEAQRVI sequence was exclusive to *S. richteri*. Why is this apparently Solr2 phenotype detected in the *S. invicta* population and at such a low incidence? Could this represent a cryptic species within the *S. invicta* range? While a possibility, it is more likely that inter-species gene flow occurred during the incipient introduction period for both species into the U.S.A. *S. richteri* and *S. invicta* were introduced through the Mobile, Alabama, port around 1918 and 1935, respectively (1), and would have been afforded an opportunity to hybridize. Indeed, we suggest that because a fertile hybrid forms readily between *S. invicta* and *S. richteri* in the U.S.A (2), they are functionally a single species in North America. Evidence to support this notion is found in Tennessee, where the two parent species were sympatric decades ago but are almost entirely hybrid today (8; Oliver et al., In Press)^1^. Gene flow is not restricted or dead-ended once the hybrid forms. The hybrid form breeds equally successfully with other hybrids and both “parent” species (34). Cohen and Privman (34) offer several possibilities for the ready hybridization of *S. invicta* and *S. richteri* observed in the U.S.A., including sympatry in the U.S.A. The specific ants introduced into the U.S.A. were genetically compatible with each other, and the introduced ants were released from various exogenous factors that prevented hybridization in the native range [e.g., diet changes, parasite infection (like *Wolbachia*)], but most likely geographic separation was removed as a species separation variable. *S. richteri* occupies the wetlands northeast of Buenos Aires, Argentina, and eastward into Uruguay, whereas *S. invicta* is native to the Pantanal area of Brazil (type specimens from Cuiaba, Brazil) and south into northern Argentina (it is found 2,690 km from Cuiaba, Brazil, in Buenos Aires, Argentina).

Interestingly, a class of compound called tyramides have been found in males from all ant species thus far examined in the Myrmicinae (35). These compounds are transferred from males to females during mating and function to initiate reproductive development in the newly mated queens (36). This transfer is critical for successful colony formation and may also act as a mechanism to maintain species integrity. One would, therefore, expect that in their native ranges where the two species are allopatric, the tyramide structure would not experience selective pressure to maintain species separation. Indeed, chemical analyses have shown this to be the case (36, 37).

Finally, phylogenetic analysis of the cytochrome oxidase I gene and the mitochondrial genome sequence data were consistent with the unusual phylogeny of the long transcript Soli2 protein sequences obtained from the InvictDetect™-failed colonies. As with the long Soli2 transcript data, cytochrome oxidase I of colonies Soli_3, Soli_4, Soli_7, and Soli_10 assorted independently of *S. invicta* and *S. richteri* but have strong sequence identity with *S. richteri*. We admit that proposing the InvictaDetect™-failed ant colonies could represent a cryptic species is not sufficiently supported by our scant data. However, one could consider the possibility because these ant colonies fit the definition of cryptic. Specifically, they are classified as a single species (*S. invicta*), morphologically indistinguishable, recently diverged, separable only with chemical/molecular data, and occur in sympatry (38).

Six Soli2 paralogs occupy a contiguous 43,627-nucleotide region of the minus strand of chromosome 10, specifically the region encompassed by nucleotides 9,600,319 to 9,643,946. These paralogs appear to be derived from duplication events, which play a major role in the rapid evolution of new biological functions (39, 40). Interestingly, gene duplication is significantly (6–7-fold in humans) higher in chromosome regions near the centromere. These pericentromeric areas are considered rapidly evolving regions of the genome (41). The *S. invicta* chromosome 10 exhibits an unusually large (6–10 Mbp) submetacentric centromere with the Soli2 region occupying an area just downstream and adjacent to the centromere of this chromosome (42). Thus, the duplication of Soli2 supports the notion that peri-centromeric regions of chromosomes exhibit rapid evolution.

Ohno (40) hypothesized that gene duplication frees one form of the gene from selective pressure, permitting the accumulation of neutral mutations that may lead to a new function (43). This hypothesis has since been validated repeatedly and among varied organisms, including invertebrates responding to various environmental pressures (44–46). Paralogs have been shown to evolve more rapidly than unduplicated genes and, if they become fixed in a population, increase fitness, which is especially noted for secreted proteins (39) such as the venom 2 protein of *Solenopsis*. Indeed, this mechanism has been reported to increase the diversity and functions of snake venoms (47). Among the small group of ant colonies collected for our study, nucleotide changes of the long Soli2 venom gene were significant, resulting in drastic amino acid changes of the protein, most notably the IEAQRVL *S. richteri*-like amino-proximal sequence (aa position 21) and the loss of the cysteine at position 41, which is changed to tyrosine in colonies Soli_3, Soli_7, Soli_8, Soli_9, and Soli_10. This cysteine is the site where dimerization occurs between monomers of the Soli2 venom protein (48).

Finally, the expression pattern for Soli2 and Soli2Tr appears to be unique in the InvictDetect™-failing ant colonies. The Soli2Tr transcript and protein were produced in these colonies from locus 105193315, which did not appear to be expressed in 98.6% of the ant colonies (based on a positive InvictDetect™ response). Soli2Tr was accompanied by Soli2 from locus 105205300 most of the time. However, in the three ant colonies, only Soli2Tr was detected, Soli_2, Soli_5, and Soli_12. Additional investigation will be required to understand the basis for the differential expression patterns and why such a small number of colonies produce this truncated protein despite the gene’s presence in the genome of *S. invicta*.

## Data Availability

The datasets presented in this study can be found in online repositories. The names of the repository/repositories and accession number(s) can be found in the article/supplementary material.

## Footnotes

1 Oliver JB, Addesso KM, Valles SM, Archer RS, Youseff NN, Pandey M, et al. *Solenopsis invicta* viruses and *Kneallhazia solenopsae* in Tennessee imported fire ant (Hymenoptera: Formicidae) populations. *Environ Entomol*. (In Press)
